# Physical Features of Intracellular Proteins that Moonlight on the Cell Surface

**DOI:** 10.1371/journal.pone.0130575

**Published:** 2015-06-25

**Authors:** Vaishak Amblee, Constance J. Jeffery

**Affiliations:** Department of Biological Sciences, University of Illinois at Chicago, MC567, 900 S. Ashland Ave., Chicago, IL 60607, United States of America; Tulane University, UNITED STATES

## Abstract

Moonlighting proteins comprise a subset of multifunctional proteins that perform two or more biochemical functions that are not due to gene fusions, multiple splice variants, proteolytic fragments, or promiscuous enzyme activities. The project described herein focuses on a sub-set of moonlighting proteins that have a canonical biochemical function inside the cell and perform a second biochemical function on the cell surface in at least one species. The goal of this project is to consider the biophysical features of these moonlighting proteins to determine whether they have shared characteristics or defining features that might suggest why these particular proteins were adopted for a second function on the cell surface, or if these proteins resemble typical intracellular proteins. The latter might suggest that many other normally intracellular proteins found on the cell surface might also be moonlighting in this fashion. We have identified 30 types of proteins that have different functions inside the cell and on the cell surface. Some of these proteins are found to moonlight on the surface of multiple species, sometimes with different extracellular functions in different species, so there are a total of 98 proteins in the study set. Although a variety of intracellular proteins (enzymes, chaperones, etc.) are observed to be re-used on the cell surface, for the most part, these proteins were found to have physical characteristics typical of intracellular proteins. Many other intracellular proteins have also been found on the surface of bacterial pathogens and other organisms in proteomics experiments. It is quite possible that many of those proteins also have a moonlighting function on the cell surface. The increasing number and variety of known moonlighting proteins suggest that there may be more moonlighting proteins than previously thought, and moonlighting might be a common feature of many more proteins.

## Introduction

Moonlighting proteins comprise a subset of multifunctional proteins that perform two or more biochemical functions that are not due to gene fusions, multiple splice variants, multiple proteolytic fragments with different functions, or promiscuous enzyme activities [1, 2]. Some of the first examples to be identified were some of the taxon specific crystallins, ubiquitous cytosolic enzymes that were adopted by a variety of species to help form the lens of the eye [3, 4]. Today, over 300 moonlighting proteins have been identified [5]. Many of the known moonlighting proteins are cytosolic enzymes, chaperones, or other proteins that exhibit a second function in other cellular locations, in other types of cells, as part of multi-protein complexes, when binding to DNA or RNA, or when the cellular concentration of a substrate, product, or other ligand changes. Data suggests, however, that there may be even more moonlighting proteins than previously thought, and increasingly more proteins with moonlighting capabilities are being uncovered.

The project described herein is focused on a sub-set of moonlighting proteins that are primarily intracellular, but perform a second biochemical function on the cell surface in at least one species. Intracellular proteins are frequently found on the cell surface during proteomics experiments. A few dozen of these intracellular/cell surface proteins have been demonstrated to have a distinct function in that location. In some pathogenic bacteria, protozoans and fungi, this extracellular function plays a key role in infection or virulence or, in the case of some nonpathogenic symbionts, in commensual interactions with a host species [6,7]. Colonization of the host requires adhesion of the bacterium or other cell type to the host, and many of these proteins have been shown to bind to proteins in the extracellular matrix or directly to host cells while some play other roles in invasion of host tissues.

The goal of this project is to consider the biophysical features of intracellular/cell surface moonlighting proteins to determine whether they have shared characteristics or defining features that might suggest why these particular proteins were adopted for a second function on the cell surface, or if these proteins are more likely to simply resemble typical intracellular proteins. The latter might suggest that many other normally intracellular proteins found on the cell surface during proteomics experiments might also have a moonlighting function in that location. We have identified 30 types of proteins that have different functions inside the cell and on the cell surface. Some of these proteins are found to moonlight on the surface of multiple species, sometimes with different extracellular functions in different species, so there are a total of 98 proteins in the study set.

## Methods

### Selection of proteins

The proteins in this study were selected with the criterion that the proteins have a biochemical function inside the cell and a second biochemical function on the cell surface. The proteins were identified by searching the literature for experimental evidence that each protein is performing a function on the surface, and is not just observed to be present on the surface.

From our analysis of the literature, we identified 30 different types of proteins, some which are found to moonlight with intracellular and cell surface functions in multiple organisms, for a total of 98 total proteins in the study. The FASTA sequence (the primary amino acid sequence) for each protein was obtained through the NCBI database (National Center for Bintechnology Information, http://www.ncbi.nlm.nih.gov), and the three-dimensional structures of each protein, when available, were obtained through the Protein Data Bank (www.rcsb.org, PDB) [8].

### Programs used

Protparam (http://web.expasy.org /) [9] was used to calculate isoelectric point (pI), amino acid composition, aliphatic index, and GRAVY (grand average of hydropathy) score [10].

The CATH Protein Structure Classification Database [11] was used to identify the types of three-dimensional folds found in the proteins. For each of the 30 types of proteins in our study set, if there is a protein in our set with a X-ray crystal structure in the Protein Data bank, that protein was selected as the representative of that type of protein. If there was no structure for a type of protein in our list, the protein that has the highest sequence identity to a protein in the PDB was chosen as the representative for that type of protein. The CATH database was then searched using the FASTA sequence for each representative protein.

UIPred [12,13] was used to predict intrinsically disordered regions of each protein. The UIPred server (iupred.enzim.hu) was used to analyze the amino acid sequence of each protein. Amino acids that scored above 0.5 were noted as being in potentially disordered regions.

SignalP, (cbs.dtu.dk/services/SignalP/) [14] and Psort, (http://psort.hgc.jp/) [15–17] were used to identify potential signal sequences.

The UniProt database (http://www.uniprot.org/) [18] was searched using the amino acid sequence of each protein to identify Gene Ontology (GO) terms in the annotation in the categories of Process and Function for each protein (www.geneontology.org) [19]. The GO terms annotated in the UniProt database predominantly describe the intracellular functions of the proteins and were used to summarize the most common intracellular pathways or roles of the proteins.

## Results

### Selection of proteins for study (Table 1)

**Table 1:** Moonlighting Proteins Used In the Study.

Intracellular Function	Surface Function	Species	UniProt	Reference
Alcohol acetaldenyde dehydrogenase	fibronectin, laminin, and type II collagen binding	*Enteamoeba histolytica*	Q24803	[20]
Alcohol acetaldehyde dehydrogenase	Listeria adhesion protein (LAP)	*Listeria monocytogenes*	Q6Q3I2	[21–24]
Aspartate ammonia lyase	plasminogen binding	*Haemophilus influenzae*	P44324	[25]
Alcohol dehydrogenase (ADH1)	plasminogen binding	*Candida albicans*	P43067	[26]
Bile salt hydrolase	plasminogen binding	*Bifidobacterium lactis*, *B*. *bifidum*, *and B*. *longum*	Q9KK62	[27]
Peroxisomal catalase (CTA1)	plasminogen binidng	*Candida albicans*	O13289	[26]
DnaK/Hsp70	plasminogen binding	*Bifidobacterium animalis*	Q8G6W1	[28]
DnaK/Hsp70	binding to invertase	*Lactococcus lactis*	P0A3J0	[29]
DnaK/Hsp70	plasminogen binding	*Mycobacterium tuberculosis*	H8EVI1	[30]
DnaK/Hsp70	plasminogen binding	*Neisseria meningitidis*	A9M296	[31]
Ef-Tu	attachment to human cells and mucins	*Lactobacillus johnsonii*	Q74JU6	[32]
Ef-Tu	fibronectin binding	*Mycoplasma pneumoniae*	P23568	[33]
Ef-Tu	factor H and plasminogen binding	*Pseudonomas aeruginosa*	B7V630	[34]
Enolase	plasminogen binding	*Aeromonas hydrophila*	Q8GE63	[35]
Enolase	plasminogen and laminin binding	*Bacillus anthracis*	D8H2L1	[36]
Enolase	plasminogen binding	*Bifidobacterium longum*, *B*. *bifidum*, *B*. *breve and B*. *lactisa*	B7GTK2	[27, 37]
Enolase	plasminogen binding	*Borrelia burgdorferi*	B7J1R2	[38]
Enolase	plasminogen binding	*Candida albicans*	P30575	[39]
Enolase	plasminogen binding	*Homo sapiens*	P06733	[40, 41]
Enolase	plasminogen and laminin binding	*Lactobacillus crispatus*	Q5K117	[42]
Enolase	plasminogen and laminin binding	*Lactobacillus johnsonii*	A3F8V9	[42]
Enolase	fibronectin binding	*Lactobacillus plantarum*	Q5NJY7	[43]
Enolase	plasminogen binding	*Leishmania mexicana*	Q3HL75	[44]
Enolase	plasminogen binding	*Neisseria meningitidis*	E0N8L2	[31]
Enolase	plasminogen binding	*Onchocerca volvulus*	Q7YZX3	[45]
Enolase	fibronectin binding	*Paracoccidioides brasiliensis*	A5JQI1	[46]
Enolase	plasminogen binding	*Rattus norvegicus*	Q5BJ93	[47]
Enolase	plasminogen binding	*Schistosoma bovis*	B2LXU1	[48]
Enolase	plasminogen and laminin binding	*Staphylococcus aureus*	E5R9G0	[42, 49]
Enolase	plasminogen binding	*Streptococcus anginosus and S*. *oralis*	E7GW07	[50]
Enolase	plasminogen binding	*Streptococcus mutans*	C6SQ43	[51]
Enolase	plasminogen binding	*Streptococcus pneumoniae*	H8LG96	[42, 52, 53]
Enolase	plasminogen binding	*Streptococcus pyogenes*	A3F8V6	[42, 54]
Enolase	plasminogen and fibronectin binding	*Streptococcus suis*	C6GGT7	[55]
Fructose 1,6-bisphosphate aldolase	plasminogen binding	*Candida albicans*	C4YHS0	[26]
Fructose-1,6-bisphosphate aldolase	adhesin	*Neisseria meningitidis*	F0N9L0	[56]
GAPDH	plasminogen binding	*Bacillus anthracis*	Q81X74	[57]
GAPDH	plasminogen, fibronectin and laminin binidng	*Candida albicans*	Q5ADM7	[26, 58]
GAPDH	NAD ribosylating activity	*Escherichia coli*	Q0TH49	[59]
GAPDH	plasminogen binding	*Lactobacillus crispatus*	D5H2B9	[60]
GAPDH	binds mucin and Caco-2 cells	*Lactobacillus plantarum*	F9UM10	[61]
GAPDH	binds invertase	*Lactococcus lactis*	F2HK64	[29]
GAPDH	binds mucin	*Mycoplasma genitalium*	J7HIM3	[62]
GAPDH	adhesin	*Neisseria meningitidis*	C6S993	[63]
GAPDH	fibronectin, laminin, and type I collagen binding	*Paracoccidioides brasiliensis*	Q8X1X3	[64]
GAPDH	transferrin-binding protein and plasminogen binding	*Staphylococcus aureus and S*. *epidermidis*	D9RFF4	[65]
GAPDH	plasminogen binding	*Streptococcus anginosus and S*. *oralis*	S6AWM2	[50]
GAPDH	plasminogen binding	*Streptococcus—group A*	Q1J8I3	[66]
GAPDH	plasminogen binding	*Streptococcus agalactiae*	Q9ALW2	[67]
GAPDH	plasminogen binding	*Streptococcus pneumoniae*	Q97NL1	[68]
GAPDH	fibronectin binding and binds uPAR/CD87 receptor on human cells	*Streptococcus pyogenes*	B5XJR2	[69, 70]
GAPDH	plasminogen binding	*Streptococcus suis*	Q3Y454	[71]
GAPDH	fibronectin, plasminogen, and collagen binding	*Trichomonas vaginalis*	Q27820	[72]
Glucose 6-phosphate isomerase	laminin and collagen I binding	*Lactobacillus crispatus*	K1MPC4	[73]
Glutamine synthetase	plasminogen binding	*Bifidobacterium lactis*, *B*. *bifidum*, *and B*. *longum*	C2GUH0	[28]
Glutamine synthetase	fibronectin, laminin, collagen I and plasminogen binding	*Lactobacillus crispatus*	D5GYN9	[73]
Glutamine synthetase	plasminogen and fibronectin binding	*Mycobacterium tuberculosis*	H8ESK0	[30]
Histone H1	thyroglobulin receptor	*Mus musculus*	P43274	[74]
Hsp60/GroEL	cytotoxic activity	*Aggregatibacter actinomycetemcomitans*	C9R2H0	[75]
Hsp60/GroEL	adhesin	*Chlamydiae pneumoniae*	P31681	[76]
Hsp60/GroEL	adhesin	*Clostridium difficile*	Q9KKF0	[77]
Hsp60/GroEL	adhesin to glycosphingolipids	*Haemophilus ducreyi*	P31294	[78, 79]
Hsp60/GroEL	adhesin	*Helicobacter pylori*	Q8RNU2	[80, 81]
Hsp60/GroEL	adhesin	*Histoplasma capsulatum*	P50142	[82]
Hsp60/GroEL	receptor for HDL	*Homo sapiens and Rattus rattus*	P10809	[83]
Hsp60/GroEL	adhesin, binds mucin	*Lactobacillus johnsonii*	F7SCR2	[84]
Hsp60/GroEL	binds invertase	*Lactococcus lactis*	F2HIT2	[29]
Hsp60/GroEL	adhesin	*Legionella pneumophila*	B2C318	[85]
Hsp60/GroEL	adhesin	*Listeria*	Q8KP52	[23]
Hsp60/GroEL	adhesin	*Mycobacterium tuberculosis*	H8EVS5	[86]
Hsp60/GroEL	adhesin	*Plesiomonas shigelloides*	Q1EQW2	[87]
Hsp60/GroEL	adhesin	*Salmonella typhimurium*	F5ZZ81	[88]
Inosine 5'-monophosphate dehydrogenase	plasminogen binding	*Staphylococcus aureus*	D6SDD0	[89]
Malate synthase	fibronectin and laminin binding	*Mycobacterium tuberculosis*	E2T9U8	[90]
Ornithine carbamoyltransferase	fibronectin binding	*Staphylococcus epidermidis*	T0BSW2	[91]
Pyruvate dehydrogenase (E1 beta subunit, PDH-B)	fibrinogen binding.	*Mycoplasma pneumoniae*	E1QCD9	[33]
Peroxiredoxin	plasminogen binding	*Neisseria meningitidis*	J8V2K1	[31]
6-phosphofructokinase	binding to invertase	*Lactococcus lactis*	F2HMQ4	[29]
6-phosphofructokinase	plasminogen binding	*Streptococcus oralis*	E6KMA1	[50]
Pyruvate-ferredoxin oxidoreductase (PFO)	adhesin	*Trichomonas vaginalis*	Q27089	[92]
6-phosphogluconate dehydrogenase	adhesin	*Streptococcus pneumoniae*	Q97SI6	[93]
Phosphoglycerate kinase	plasminogen bindng	*Candida albicans*	P46273	[26]
Phosphoglycerate kinase	plasminogen binding	*Streptococcus agalactiae* S8XU00	S8XU00	[94]
Phosphoglycerate kinase	plasminogen binding	*Streptococcus anginosus and S*. *oralis*	E7GZG8	[50]
Phosphoglycerate kinase	plasminogen binding	*Streptococcus pneumoniae*	J1RST3	[95]
Phosphoglycerate mutase	plasminogen binding	*Bifidobacterium lactis*, *B*. *bifidum*, *and B*. *longum*	S3DNJ2	[27]
Phosphoglyceromutase	plasminogen binidng	*Candida albicans*	P82612	[26]
Phosphoglycerate mutase	plasminogen binding	*Streptococcus anginosus and S*. *oralis*	E6IYJ0	[50]
Pyruvate kinase	binds invertase	*Lactococcus lactis*	F2HMQ3	[29]
Ribonucleotide reductase subunit 2	plasminogen binding	*Staphylococcus aureus*	Q7A6T1	[89]
Superoxide dismutase	adhesin	*Mycobacterium avium*	P47201	[96, 97]
Superoxide dismutase	Adhesion	*Mycobacterium tuberculosis*	H8F202	[97]
Transcription elongation factor (TEF1)	plasminogen binding	*Candida albicans*	C4YDJ3	[26]
Triose phosphate isomerase	adhesin	*Paracoccidioides brasiliensis*	Q96VN5	[98]
Triose phosphate isomerase	adhesin	*Staphylococcus aureus*	D9RMW0	[99]
Triose phosphate isomerase	plasminogen binding	*Streptococcus anginosus and S*. *oralis*	E6J203	[50]
Thiol-specific antioxidant protein (TSA1)	plasminogen binding	*Candida albicans*	C4YNZ5	[26]

The proteins in this study [20–99] were selected with the criteria that each protein has a biochemical function inside the cell and at least one different biochemical function on the cell surface. The proteins were identified by searching the literature for experimental evidence that each protein is performing a biochemical function on the cell surface. Proteins that were only observed to be present on the cell surface and have not had a function identified in that location were not included in the study. This latter requirement removes proteins that may have been observed on the cell surface as potential false-positives in proteomics experiments. In addition, for this study, proteins that are secreted but not attached to the cell surface were not included. From our analysis of the literature, we identified 30 different types of proteins, some which are found to moonlight with intracellular and cell surface functions in multiple organisms, for a total of 98 total proteins in the study.

### Species and types of organisms

Most of the proteins in the study are from bacteria. The bacterial species represented include typical Gram-positive and Gram-negative species, as well as mycobacteria, spirochetes, and mycoplasma. Most of the species represented are pathogenic, but some are considered “pro-biotic”, or members of the normal gut biota. A few of the proteins are found to moonlight in single-celled eukaryotic organisms, including protozoa and yeast fungi, or in multicellular eukaryotes, including mammals and worms.

### Intracellular Functions

The majority of the proteins in the study are metabolic enzymes. Alll six Enzyme Commission (EC) groups are represented. Of the 23 types of enzymes, ten are in EC group 1 (oxidoreductases) and five are in group 2 (transferases). Most of the enzymes are ubiquitous or at least found in many species and function in central pathways in metabolism, including glycolysis, the citric acid cycle, the pentose phosphate pathway, or in nucleotide or amino acid metabolism. Other intracellular/cell surface moonlighting proteins include chaperones (heat shock protein 60 Hsp60/GroEL, heat shock protein 70 Hsp70/DnaK), as well as a protein synthesis elongation factor (Ef-Tu, elongation factor Tu), a transcription elongation factor (transcription elongation factor 1, TEF1), a thiol specific antioxidant protein (TSA1), and a histone (H1).

### Extracellular functions

**Fig 1:**
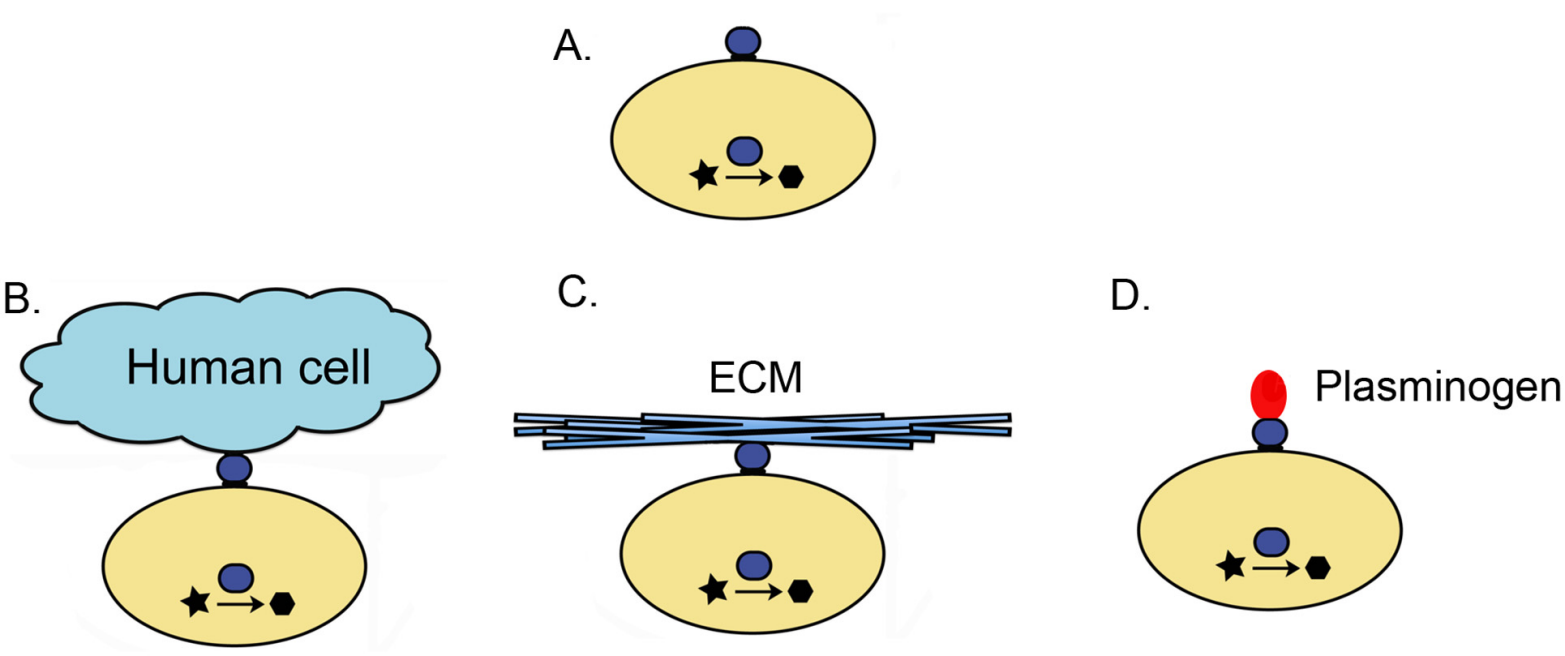
An increasing number of intracellular enzymes, chaperones, and other proteins are being found on the cell surface where they perform other functions. A single protein can have a function inside the cell, for example and enzyme that converts a substrate (star) to a product (hexagon), and also be found on the cell surface (A). Some of these proteins moonlight as adhesins for binding to host cell surface proteins (B) or to extracellular matrix (C, ECM) and play a role in infection and virulence. Other proteins bind to the zymogen plasminogen and enable its conversion to plasmin, a broad specificity protease (D). The active protease is then used as an aide to degrade and invade host tissues.

The majority of the intracellular/cell surface moonlighting proteins function on the cell surface in binding to extracellular matrix, as an adhesin to attach to host cells, or as a cell surface receptor for a soluble protein (Fig 1). For those proteins described as adhesins in Table 1, the specific host protein on the host cell surface has been identified in only a few cases. *Streptococcus pyogenes* glyceraldehyde 3-phosphate dehydrogenase (GAPDH) binds to the uPAR/CD87 receptor on human cells [70]. *Haemophilus ducreyi* Hsp60/GroEL binds to glycosphingolipids [78, 79]. On the surface of *Listeria monocytogenes*, alcohol acetaldehyde dehydrogenase is used to bind to a moonlighting Hsp60 on the surface of mammalian host cells, an interesting example of a moonlighting protein interacting with another moonlighting protein [22, 23].

Many of the bacterial moonlighting proteins bind to structural components of the host extracellular matrix, including fibronectin, laminin, and/or collagen, or to mucin, a component of the mucosal epithelial lining. These interactions enable a physical attachment to the host, whether it is for a pathogen invading host tissues or a commensual gut bacterium establishing a more symbiotic relationship with a mammalian host. When *Lactococcus lactis* Hsp60/GroEL, DnaK/Hsp70, GAPDH, pyruvate kinase and 6-phosphofructokinase bind yeast Invertase, a hyperglycosylated cell surface protein, the interaction may also assist in a symbiotic relationship between the bacterium and the yeast [29].

Several of the moonlighting proteins in the study are receptors for soluble proteins. Human and mouse Hsp60/GroEL are cell surface receptors for high density lipoprotein (HDL) [83], and mouse histone H1 serves as a thyroglobulin receptor to mediate thyroglobulin endocytosis [74]. Staphylococcal GAPDH serves as a transferrin binding protein to acquire iron from the host [65]. Binding to the soluble protein plasminogen aids in invasion of host tissues by many pathogens. Plasminogen is a precursor to plasmin, which is a serine protease present in blood that helps break down fibrin clots [100]. When an invading pathogen uses a surface moonlighting protein to bind plasminogen from the host, the plasminogen can be converted to plasmin, the active form of the protease, by tissue-type plasminogen activator (tPA) and urokinase. The plasmin that is now attached to the surface of the invading organism can be used as a general protease to degrade host extracellular matrix and basement membrane, thereby facilitating migration through tissues.

We note that in many of the published reports about the proteins in our study, the intention was to determine if the species being studied had any surface proteins that bind to a specific target protein (plasminogen, fibronectin, etc.) Each of these intracellular/cell surface proteins may also have other functions on the cell surface in addition to the ones that have been identified to date, for example some of the proteins that are known to bind to plasminogen might also bind to other host proteins.

### Physical features

The primary amino acid sequence for each protein and the three-dimensional structures of each protein, when available, were studied using bioinformatics tools in order to ascertain general shared characteristics or defining features of these proteins as a set, be they structural features, sequence motifs, or biochemical properties. Any trends, patterns, or generalizations that we discover among these moonlighting proteins could aid in the identification of other proteins that may have a moonlighting function on the cell surface.

#### Signal sequences

In general, most proteins targeted to the cell surface contain an N-terminal signal peptide, but many of these intracellular/cell surface moonlighting proteins have been found to lack a signal sequence. SignalP [14] and Psort [15–17] were used to look for the presence of a signal sequence for targeting to the plasma membrane for the study set of 98 proteins. None of the proteins contain a signal peptide.

#### Molecular weight

**Fig 2:**
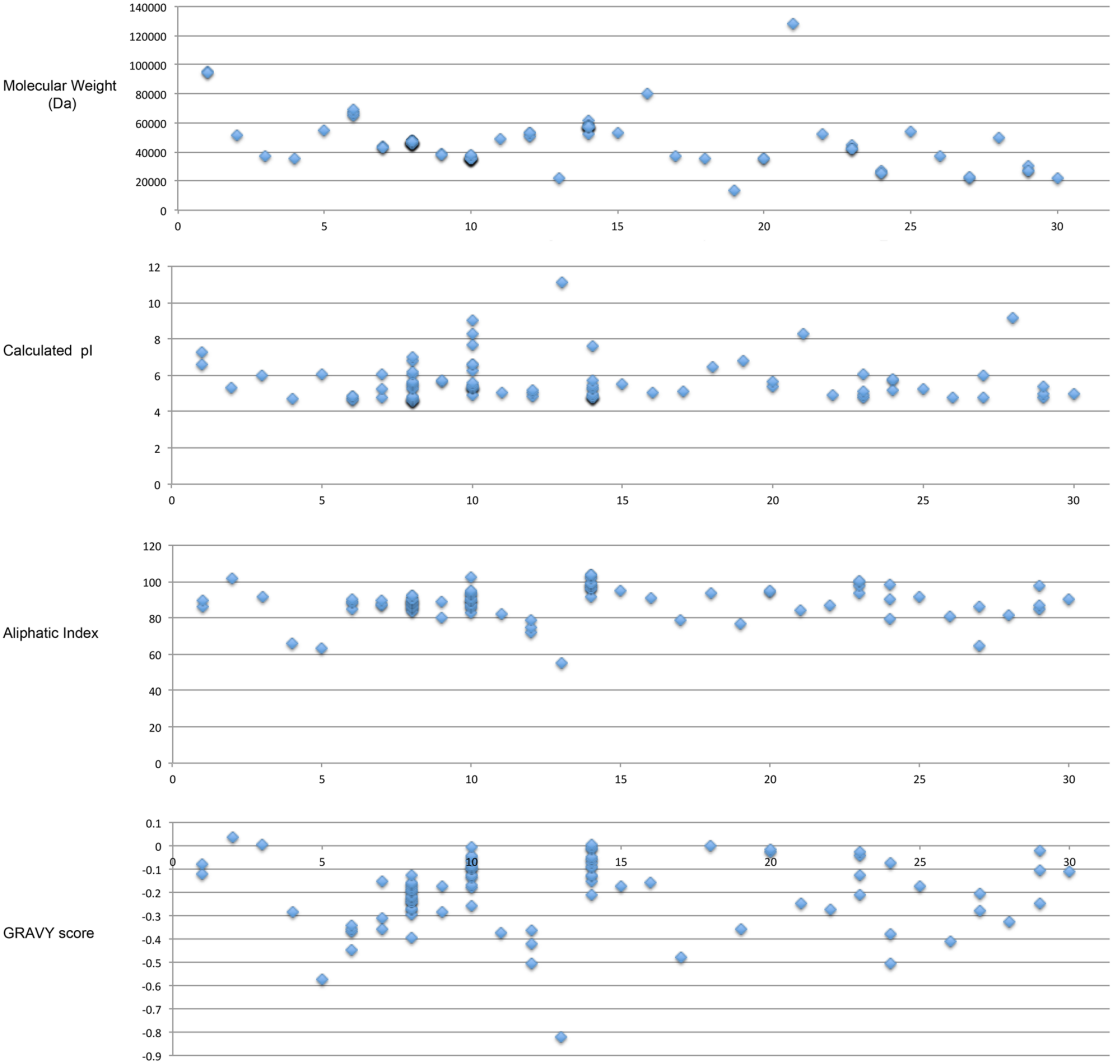
Physical features of intracellular proteins that moonlight on the cell surface. Protein molecular weight (A), calculated pI (B), aliphatic index (C), and GRAVY score (D) are shown for each type of protein. For some types of proteins, more than one score is shown because the protein is found to moonlight in more than one organism, and the score was determined for each protein in each organism in which it moonlights. The type of enzyme is indicated by number on the x-axis: 1. Alcohol acetaldehyde dehydrogenase. 2. Aspartase. 3. Alcohol dehydrogenase. 4. Bile salt hydrolase. 5. Peroxisomal catalase. 6. DnaK. 7. Ef-Tu. 8. Enolase. 9. Fructose 1,6-bisphosphate aldolase. 10. GAPDH. 11. Glucose 6-phosphate isomerase. 12. Glutamine synthetase. 13. Histone H1. 14. Hsp60/GroEL. 15. IMPDH. 16. Malate synthase. 17. Ornithine carbamoyl transferase. 18. Pyruvate dehydrogenase. 19. Peroxiredoxin. 20. 6-phosphofructokinase. 21. Pyruvate-ferredoxin oxidoreductase. 22. 6-phosphogluconate dehydrogenase. 23. Phosphoglycerate kinase. 24. Phosphoglyceromutase. 25. Pyruvate kinase. 26. Ribonucleotide reductase. 27. Superoxide dismutase. 28. Transcription elongation factor. 29. Triose phosphate isomerase. 30. Thiol specific antioxidant protein TSA1. Calculations were performed with ProtParam [9].

The individual proteins of each specific type in our study varied very little in molecular weight regardless of species of origin (Fig 2a). Most of these moonlighting proteins are within the range of 200–600 amino acids. The longest were alcohol acetaldehyde dehydrogenase (866–870 amino acids) and pyruvate-ferredoxin oxidoreductase (1157 amino acids). Brocchieri and Karlin [101] found through a study of 5 eukaryotic genomes and 67 bacterial genomes that the median length of proteins in eukaryotes is 361 amino acids and the median length is only 267 amino acids in bacteria. Other studies of many genes from a genome or from whole genomes found similar averages for protein length [102]. Interestingly, most of the proteins in our study set are significantly longer than these median sizes. The eukaryotic proteins in our study contain from 196 to 1157 amino acids, with eight protein types containing over 400 amino acids, three types near the observed median length (between 330 and 360 amino acids) and 4 protein types containing fewer than 250 amino acids. The bacterial proteins ranged from 122 amino acids (peroxiredoxin) to 866 amino acids (acetaldehyde dehydrogenase) in length. Only three types of proteins contain fewer than the median of 267 amino acids observed by Brocchieri and Karlin (peroxiredoxin, superoxide dismutase, and phosphoglycerate mutase), and one protein was near the median length (triose phosphate isomerase (TPI), with 252 amino acids in *Streptococcus* TPI and 283 amino acids in *Staphylococcus* TPI), twenty of the bacterial protein types in the study contain over 300 amino acids.

#### Theoretical pI

The calculated isoelectric points (pI) have been found to differ among proteins that localize to different sub-cellular locations [103]. Proteins are generally least soluble near their isoelectric points, which means that a protein’s pI needs to be different from its environmental pH for the protein to be adequately soluble. Cytoplasmic pH is usually around 7, so proteins with a pI greater than or less than this pH are favored. The calculated pIs for known cytosolic proteins have been found to center around 5.5 [103]. The pI’s of most of the proteins in our study lie between 4.5–6.5 (Fig 2b), which is typical of cytosolic proteins.

#### Aliphatic index

The aliphatic index is a measure of the relative volume occupied by aliphatic side chains—alanine, valine, isoleucine, and leucine. A higher aliphatic index is an indicator of higher thermostability, and also an indicator of solubility in a cell when the protein is overexpressed [104]. Most cytosolic enzymes have aliphatic indexes around 80–100, as did most of the proteins in this study set (Fig 2C) [104]. However, Histone H1 (55.3), which is a nuclear protein, CTA1 peroxisomal catalase (64), bile salt hydrolase (66), glutamine synthase (72–79), peroxiredoxin (77), and one of the superoxide dismutases (65) had aliphatic indexes below 80.

#### GRAVY score

The Grand Average of Hydropathy (GRAVY) for a protein is calculated as the sum of hydropathy values of all the amino acids, divided by the number of residues in the sequence [10]. More hydrophobic/non-polar residues are given more positive values, whereas more polar/ionic residues are given more negative values. The overall GRAVY scores for the proteins in this study were negative or zero for all except aspartase, which was slightly above zero at 0.036 (Fig 2d). These scores are typical for soluble proteins. None of the proteins had sequences of hydrophobic amino acids sufficiently long enough to indicate the presence of a transmembrane domain.

Intrinsically disordered regions of proteins allow many different conformational states, and in many proteins enable highly specific interactions with multiple binding partners [105]. Intrinsically disordered regions also may be more tolerant of mutations than more structured domains, and thereby might provide the material for evolution of an additional function. We studied the amino acid sequences of the intracellular/cell surface moonlighting proteins using UIPred to identify potential regions of disorder [12, 13]. Analysis using UIPred identified only very short regions of most of the proteins that scored above 0.5 and are most likely surface loops. Only Histone H1 contained longer intrinsically disordered regions. These results suggest that most of the intracellular/cell surface moonlighting proteins in this study set are not likely to be the type of moonlighting proteins that interact with multiple proteins by folding into different conformations with different binding partners.

#### Type of three-dimensional fold

Different protein folds vary in their stability and their tolerance of amino acid sequence substitutions, insertions and deletions. In addition, some folds might make better “scaffolds” for evolution of new functions, because of additional stability, for example. The CATH protein structure classification system is a hierarchical classification of protein domains [11]. Of the 30 different types of proteins in our list, there were 58 CATH domain classifications represented (some proteins contained multiple domains). Thirty-nine domains were in Class 3 (containing a significant amount of both alpha-helical and beta sheet secondary structure elements), 13 were in class 1 (mostly alpha-helical), and only 6 domains were in Class 2 (mostly beta sheet). Consistent with the IUPred results, no domains were classified as having very little secondary structure (Class 4). The CATH Architectures (arrangements of secondary structures) that were represented most often include 1.10 or mainly alpha/orthogonal bundle, 1.20 mainly alpha/up-down bundle, 3.20 alpha-beta/alpha-beta barrel, 3.30 alpha-beta/2-layer sandwich, 3.40 alpha beta/3-layer (aba) sandwich. Two topology or fold groups were represented at least six different proteins: 3.20.20 TIM barrel and 3.40.50 Rossmann fold. TIM barrels and alpha/beta sandwiches (which includes the Rossman fold) are two of the most common architectures for proteins in general.

## Discussion

A goal of this bioinformatics analyses was to determine if there are any characteristics or trends that could define and help identify intracellular/cell surface moonlighting proteins. Although a variety of intracellular proteins (enzymes, chaperones, etc.) are observed to be re-used on the cell surface, for the most part they seem to be typical intracellular proteins. Other than the few exceptions mentioned above, they do not exhibit extremes in calculated pI, disorder, stability, or other characteristics. The types of three-dimensional folds are diverse and common among many proteins.

The question remains as to why and how these proteins obtained a second function. Like many of the other known moonlighting proteins, most of the proteins in this study are ubiquitous enzymes in central metabolism or ubiquitous chaperone proteins. They are likely to have been adopted for a second function because organisms evolve by utilizing and building upon components they already possess, and these proteins are available in many organisms. Many of the moonlighting proteins in this study, as well as many described elsewhere [1–7, 106, 107], are essential housekeeping proteins. These proteins first arose billions of years ago and are expressed in many species and cell types, making them available targets for organisms to modify and use to develop a new function.

A new binding function can result if a protein’s structure is modified to create a new binding site on the protein surface, and binding to another protein is the key characteristic of the second function of most of the proteins in this study. Modification of a short amino acid sequence could be sufficient to form a new protein-protein interaction site. In general, proteins appear to contain many more amino acids than are required to form their active site, leaving a lot of surface amino acids that are not involved in the first function and are therefore not under as much selective pressure. For example, the active site amino acid residues of the glycolytic enzyme phosphoglucose isomerase have shown to be highly conserved in over 126 species, however the solvent-exposed areas are not as carefully conserved and contain a multitude of loops, pockets, clefts and other structural features that have been modified during billions of years of evolution and could have easily developed a new binding site, yielding a new moonlighting function for this protein [108]. It is interesting to note that one trend of the proteins in this study is that they tend to be somewhat longer than the median length of a cytosolic protein. Perhaps having more amino acids than the average protein increases the probability that a small surface region can be modified by evolution to form a new protein binding site without affecting the original function of the protein.

A large number of the proteins in the study bind plasminogen on the cell surface. The binding site for plasminogen has been found in several cases to be a short lysine-containing amino acid sequence usually at the C-terminus of the protein, although found internally in some enolases [109, 110]. Addition of a plasminogen binding site by replacement of a few surface or C-terminal residues with lysines is just the kind of modification that could add a second function without significant changes to the overall protein structure or original function. It is also possible that proteins that already contain lysines in appropriate places could be adopted for a second function if they were to be expressed in a new location, for example on the cell surface. This adoption of proteins for a new function by changes in expression without significant modification of the protein structure is how several ubiquitous enzymes became taxon specific crystallins [111].

Another possible reason for the increased sequence length is suggested by an observation by Ghosh and Dill that longer proteins tend to be more stable than shorter proteins [112]. Because the moonlighting function of each of the proteins in our study involves being displayed on the cell surface, in a harsher environment than in the cell cytoplasm, proteins with an above average level of protein stability may have been selected for this function. Another correlation with protein length is the degree of amino acid sequence conservation, with conserved proteins tending to be longer [113]. This is consistent with many of the moonlighting proteins in our list being conserved proteins with original functions in central metabolism.

These moonlighting intracellular/cell surface proteins not only need a method to interact with another protein, but also they need a mechanism to be transported across the cell membrane and a mechanism to become attached to the cell surface. None of the proteins have been found to possess a signal peptide for targeting to the cell membrane or a sequence motif for attachment to the cell surface, for example the LPXTG motif that is involved in attachment to the cell surface in Gram-positive bacteria [114]. How these intracellular proteins end up located outside of the cell and attached to the cell surface is an active area of inquiry in this field.

Many other intracellular proteins have also been found on the surface of bacterial pathogens and other organisms in proteomics experiments. It is quite possible that they also have a moonlighting function on the cell surface. The increasing number and variety of known moonlighting proteins suggest that there may be more moonlighting proteins than previously thought, and moonlighting might be a common feature of many more proteins.
